# Development and validation of a questionnaire (GHOST) to assess sudden, unexplained communication exclusion or “ghosting"

**DOI:** 10.1016/j.heliyon.2023.e17066

**Published:** 2023-06-08

**Authors:** Haitham Jahrami, Zahra Saif, Wen Chen, Mai Helmy, Hadeel Ghazzawi, Khaled Trabelsi, Gabriel Natan Pires, Nicola L. Bragazzi, Seithikurippu R. Pandi-Perumal, Mary V. Seeman

**Affiliations:** a Government Hospitals, Manama, Bahrain; b Department of Psychiatry, College of Medicine and Medical Sciences, Arabian Gulf University, Manama, Bahrain; c Department of Child Psychology, The Children's Hospital, Zhejiang University School of Medicine, National Clinical Research Center for Child Health, National Children's Regional Medical Center, Hangzhou, Zhejiang, China; d Psychology Department, College of Education, Sultan Qaboos University, Muscat, 123, Oman; e Psychology Department, Menoufia University, Shebin El-Kom, 32511, Egypt; f Nutrition and Food Technology Department, Agriculture School, The University of Jordan, P. O. Box 11942, Jordan; g High Institute of Sport and Physical Education of Sfax, University of Sfax, Sfax, 3000, Tunisia; h Research Laboratory: Education, Motricity, Sport and Health, EM2S, LR19JS01, University of Sfax, Sfax, 3000, Tunisia; i Federal University of São Paulo, Brazil; j Human Nutrition Unit, Department of Food and Drugs, University of Parma Medical School, Parma, Italy; k Division of Research and Development, Lovely Professional University, Phagwara, Punjab, 144411, India; l Saveetha Medical College and Hospitals, Saveetha Institute of Medical and Technical Sciences, Saveetha University, Chennai, Tamil Nadu, India; m Department of Psychiatry, University of Toronto, Canada

**Keywords:** Cyberpsychology, Icing, Psychometrics, Simmering, Social media, Social networking

## Abstract

The topic of “ghosting” as a method of terminating a relationship has been discussed in both popular media and academic circles. Although research on this issue is scarce, the concept has acquired popularity and gained scholarly attention. A reliable and valid measure of this phenomenon does not, however, exist. GHOST (The Ghosting Questionnaire) was designed and psychometrically tested to explore ghostee experiences. A total of 811 adults participated in an online survey to test this instrument. It was developed based on a thorough analysis of research on the topic of ghosting using a phenomenological qualitative method to identify ghosting domains and generate questionnaire items. In terms of content validity and construct validity, the final version of the measure was found to be satisfactory. GHOST was found to have adequate internal consistency - scores of 0.74, 0.74, and 0.80, indicating acceptable Cronbach's alpha, McDonald's omega, and ordinal's alpha coefficients, respectively. Factor analyses found the GHOST questionnaire to be a valid and reliable instrument that can be used for screening ghosting experiences and for designing community-based distress prevention and intervention programs. A dynamic fit index (DFI) cutoffs approach was also used and showed robust fitting.

## 1 Introduction

Technological advances are altering lifestyles and changing interaction and communication practices. The term *ghosting*, alias *simmering* or *icing*, describes a practice in which all communication with another person is terminated without any warning or justification. For no stated reason, the lines of communication are abruptly cut [1]. The term was coined in 2000 in reference to partner behavior in dating and romantic relationships [2]. As social media and online apps increased over the last two decades, ghosting has become more prevalent. The term is now being used to refer more broadly to communications between friends, family members, employers/employees [3]. Ghosting takes place in the context of personal relationships, motivated by the wish to discontinue contact but, at the same time, avoid the discomfort of a face-to-face traditional break-up scene [2]. The goal of persons who do the ghosting is to protect themselves from the distress of delivering bad news, but they do not consider the impact of silence on the ghostee. Psychologists have described ghosting as a passive-aggressive form of emotional abuse or cruelty that negatively affects the person on the receiving end [2,4].

Knowledge about ghosting, a media favorite, comes from information collected by journalists, much like information about other trending cyberpsychology subjects, such as “breadcrumbing,” “nomophobia,” “orbiting” [1,[5], [6], [7], [8]]. Little scientific research has addressed the topic [4,9] and publications about motives and vulnerabilities are scarce. Most studies focus on the prevalence or consequences of ghosting victimization [1,5,10]. Currently, there is little empirical evidence regarding the characteristics of persons who have tendencies to ghost others and those persons who are most likely to become ghosting victims, which makes it challenging to create effective prevention and therapeutic strategies [6,7].

There is currently no established standard method to evaluate what is and what is not ghosting; the term has only been informally defined [1,[5], [6], [7]]. This poses a significant challenge for researchers and practitioners concerned about its negative effects on mental health and well-being [4,9]. According to theoretical frameworks of the psychological impact of perceived social exclusion, being ghosted can result in feelings of rejection, anxiety, and lowered self-esteem [2,4]. It is therefore important to develop a reliable and valid questionnaire about ghosting for purposes of both research and clinical practice. Such a measure would lead to a precise definition of the phenomenon of ghosting and the specifics of its impact on individuals.

Our measure of ghosting is based on the Shannon-Weaver communication model [11]. This model explains how information is conveyed, processed, and received by individuals and groups [11]. Several concepts are included that aid in understanding and evaluating communication [12]. Sender, encoder, channel, noise, decoder, and receiver are the six main components of the model [11]. Since the model is linear, it can support a one-dimensional structure (i.e., communication) [11].

**Fig. 1:**
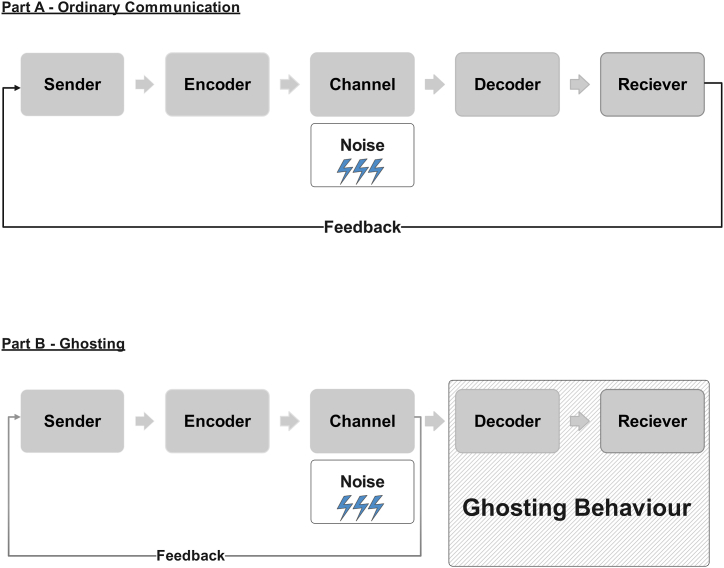
Schematic diagram of communication/ghosting based on the Shannon-Weaver model. Part A – Ordinary communication. Part B – Ghosting.

Senders (also known as information sources) are the originators of content or messages [11]. The sender can be a person, a group, or an organization that has information to be shared [11]. The encoder (also known as the transmitter) is responsible for transforming the sender's message into a suitable format for transmission. This involves changing the message into a signal or a sequence of signals deliverable across a communication channel [11]. The channel is the medium by which the encoded message is sent from sender to receiver [11]. It can be a physical medium such as air, wire, or fiber optic cable, or a virtual medium such as a digital network or radio waves [11]. Any unwanted interference or disruption that can compromise the clarity or accuracy of the message during transmission is referred to as noise. Noise, such as environmental perturbation, might be external, or internal, for instance, faults in the encoding or decoding process [11]. The decoder decrypts the message sent through the channel. The decoder turns the received signals back to the original message, understandable to the intended recipient [11]. The receiver (also known as the destination) is the intended recipient of the original message [11]. Fig. 1 provides a schematic diagram based on the Shannon-Weaver model.

In ordinary communication, a person (sender) who would like to send a message (i.e., text, an image, a video, an emoji, etc.) can use his/her mobile phone (encoder) to transmit this message using a mobile phone network (channel), which is received by the recipient's mobile phone (decoder), to be read by the receiver. Mobile network outages or impaired signal quality are possible noises that might affect communication. Receiving the message accurately is important for the receiver in order to be able to provide feedback to the sender. The circuit is illustrated in Fig. 1, Part A.

Ghosting, the phenomenon of someone abruptly breaking off all communication with another person in the context of a personal relationship can be examined using communication theory [[13], [14], [15]]. In ghosting, some elements of the communication model are interrupted that affect the decoder and the receiver. For example, a sender sends a message on a mobile phone (encoder) that is transmitted over a mobile phone network (channel). Here the message ends for reasons that are unknown to the sender. The recipient mobile phone (decoder) may have been configured to “blocked communication” or else the receiver is intentionally ignoring/deleting the messages received. The sender does not know the reason for the lack of response. The sender often assumes the reason is network noise and, therefore, attempts to recommunicate with the receiver. The only feedback received by the sender is an automatic status update from the channel (i.e., mobile phone network) in the form of a delivery receipt confirming that messages were delivered and/or read. This scenario is illustrated in Fig. 1, Part B.

The potential psychological harm of the feedback that a message to an important other has been received but intentionally ignored depends on a number of personality and relationship factors, which need to be better understood. Further research into the profile of ghosting victims and ghosting's impact on mental health and well-being awaits the development of a valid and reliable measuring instrument. This study's goal was to develop and evaluate a ghosting questionnaire (GHOST). We hypothesized that a valid questionnaire could be developed based on the Shannon-Weaver model. The questionnaire would be a useful tool for gathering information on the frequency, reasons for, and outcomes of ghosting behavior in different kinds of relationships. It could be useful in research and in the clinic. Being able to understand and quantify ghosting can help with treatment of negative effects and reveal interpersonal dynamics, communication habits, and emotional control.

## 2 Material and methods

### 2.1 Study design

Following best practice guidelines, there were three phases to the process of developing the questionnaire: item development, scale development, and scale evaluation [16].

#### 2.1.1 Phase 1: item development

Step 1 identification of the questionnaire

We first established a sound theoretical framework, the Shannon-Weaver model. This helped to identify the construct of ghosting and its constituent parts. We consulted with subject-matter specialists and read the available ghosting literature prior to Item generation. To this end, we searched the following academic databases: EBSCO (including CINHAL), PubMed/MEDLINE), PsycINFO, ScienceDirect, Scopus, and Web of Science (ISI) using the following terms: “ghosting”, “simmering”, “icing”. We identified a total of 20 relevant articles. After removing duplicates and irrelevant papers, eight useable items were obtained. The main emerging themes at this stage were: 1) Abruptly stopping communication with the other person [4,17]. 2) Not returning a call or message from the other person without providing a reason [4,17,18]. 3) Abruptly change plans without a valid justification [19]. 4) Lying about being unavailable or busy to avoid spending time with someone [5,10]. 5) Breaking off a relationship without explaining why to the other person [[4], [5], [6],^10^]. 6) Ending a conversation with the other person [4,5,10]. 7) On social media, blocking or unfriending the other person without explanation [6,7,20]. 8) Leaving online chat or group discussion without saying goodbye [4,5,10].

The second phase consisted of interviewing volunteer participants using a phenomenological method [21] for qualitative investigation. Phenomenology entails using narrative accounts of participants' encounters with the phenomenon in question [21]. To thoroughly comprehend the dimensions of ghosting based on respondents' personal experiences, three batches of semi-structured interviews were undertaken, using 5–7 anonymous participants per batch. We used the JumpChat platform [22] (free, easy to use, requires no new software, no plugins; no accounts, and has built-in privacy guarantees). Participants were crowdsourced using investigators’ social media apps. The primary inclusion criterion was willing adult participants regardless of sex, ethnic background, age, or other demographic constraints. Each interview lasted for 45–60 min.

Participants were requested to sign an informed consent form by clicking “enter meeting room” after the primary investigator had welcomed them and explained the study in the meeting link's “waiting area.” The interviews were conducted in English, and written notes were taken during the interviews. To ensure that each respondent received the same information about the study and was asked the same questions, an interview guide was followed. Three questions were asked: What is ghosting, how does it happen, and what are the basic ghosting techniques/practices? Two items for the questionnaire were generated in this way, and added to the 8 from the literature resulting in 10 intentionally unidimensional items [23]. [Note: The pronouns they/them/theirs refer to the “ghosters”].

The preliminary 10 questionnaire items.

Ghost 1 - Did you get stood up or had your plans canceled by them without being told beforehand?

Ghost 2 - Despite sending messages, you have not heard back from them.

Ghost 3 - Their reply/response messages are delayed.

Ghost 4 - Their reply/response messages are confusing and vague.

Ghost 5 - Have you been blocked or deleted from their social media apps or messaging apps.

Ghost 6 - Have you experienced trouble maintaining a conversation with them (e.g.*,* they use one-word replies, confusing emojis, short responses).

Ghost 7 - The phrase “I'm busy” is always used in their communications.

Ghost 8 - Their interest in you is inconsistent - sometimes very engaged, sometimes completely uninterested.

Ghost 9 - They don't share personal information about themselves with you.

Ghost 10 - They are not interested in meeting.

Step 2 content validity

The items' content was then evaluated for relevance by a multidisciplinary panel of nine specialists, which included two psychologists, a psychiatrist, two research methodologists, two biostatisticians, a biomedical researcher, and a therapist. A Likert-style scale was utilized, with “1″ denoting no relevance and “4″ denoting high relevance. Then, the content validity index (CVI) [24] was determined for each item. According to Lynn's guidelines, a CVI of 0.80 or higher was considered adequate [24]. In the end, all ten items were deemed pertinent enough to be added to the final instrument (CVI >0.85), which was named the Ghosting Questionnaire (GHOST).

#### 2.1.2 Phase 2: scale development

Step 3 Pre-testing questions

Through participation in a paper-and-pencil survey, a sample of 35 randomly selected adults consented to pilot GHOST. The participants' basic sociodemographic details, such as age and sex, were documented to ensure generalizability to the local population. Potential participants who confirmed a diagnosis of mental disorder were excluded after a quick screening process that included direct questions and a skip logic algorithm. The 5-point Likert scale designated responses as (1 = never, 2 = rarely, 3 = occasionally, 4 = frequently, and 5 = always). The higher the score, the more strongly the respondent agrees with the concept of “having been ghosted.” None of the participants in the pre-test found aspects of the questionnaire that needed clarification or rewriting. Less than 5 min were needed to complete the questionnaire.

##### 2.1.2.1 Step 4: survey administration and sample size determination

For this study, we mimicked a crowdsourcing platform (e.g.*,* MTurk) by soliciting responses from a large pool of participants via various instant messaging chat groups and social media announcements. In September 2022, the study was conducted utilizing an online Google form.

The number of observed variables is frequently taken into account while determining the sample size in psychometrics [25]. In this study, the lower limit of sufficient sample size was established as 10 cases or observations for each indicator variable expressed in a Likert-like scale [26]. Thus, n = q*10; where n is the sample size, and q is the number of questions. By substituting the formula, we ended up with n = 10 * 10 = 100. Guadagnoli and Velicer proposed that a minimum of 300 is necessary to observe good validation based on their simulation analysis utilizing varied sample sizes [27]. To further increase power, we aimed to recruit 400 participants following Jackson's recommendation [28].

Step 5 item reduction analysis and Step 6: extraction of factors

In using a composite scale score based on item responses, it is implicitly assumed that the scale is dominantly unidimensional. An essential component of construct validity is determining whether the item response data are or are not [29]. In assessing the dimensionality of item response data, however, there are no universally accepted methods or rules [29]. Commonly used approaches are eigenvalues, parallel analysis, and root-mean-square-error-of-approximation [29].

An exploratory factor analysis (EFA) [30] using maximum likelihood estimation (MLE), with no rotation, and Kaiser normalization [31] was carried out to investigate the construct validity of GHOST. The orthogonal and unique variance of the generated factors, which can make the interpretation of the factor structure easier, is one advantage of not applying rotation following MLE extraction [31]. The fact that the derived factors are uncorrelated and consequently have distinct explanatory power is another advantage of not applying rotation after MLE extraction. This can be helpful when trying to isolate a few fundamental causes that account for the majority of the variance in the observed variables [32].

We utilized a scree plot to display the outcomes of the parallel analysis. The eigenvalues from the factor analysis are shown graphically, from greatest to smallest, in a scree plot. The number of elements that should be retained is represented by a curve on the scree plot that first declines sharply before leveling off.

#### 2.1.3 Phase 3: scale evaluation

Step 7 tests of reliability, Step 8: tests of dimensionality, and Step 9: tests of validity

GHOST reliability was explored using several metrics, including Cronbach alpha (Cα) [33], ordinal alpha (Oα) [34], McDonald's omega (ω) coefficients [35], inter-item correlation [36], item-rests (aka item-total) correlation coefficients [37]. For Cα, Oα, and ω, we also reported coefficients of alpha if items were removed. Generally, an α/ω above 0.70 is considered acceptable [38].

An Oα, which is conceptually equivalent to Cα, is most appropriate when ordinal-type scales are used. Cα measures the correlation between a group of items based on tau-equivalence models [39]. McDonald's ω coefficient is based on a congeneric model, so it takes strength and direction into account in determining the correlation [35]. Because it does not require assumptions of unidimensionality or tau-equivalence, it is considered a more robust method for estimating internal consistency [35].

The Gaski approach for assessing convergent validity was used in this research [40]. It involved cross-correlating each item in the questionnaire with the remaining items to ensure that they are measuring the same construct [40].

To test the dimensionality and validity of the GHOST questionnaire, a Confirmatory Factor Analysis (CFA) [41] was conducted. A CFA was conducted on the remaining sample that was not used for EFA (***i.e.,*** participants in both EFA and CFA are independent). CFA was used to verify the factor structure obtained in previous steps (5 and 6).

We calculated the following fit indices: absolute indices, discrepancy indices, and residual-based indices: Chi-squared and maximum-likelihood [ML] chi-squared are absolute indices or discrepancy indices [42]. Based on the Bayesian information criterion (BIC), when a finite collection of models is considered, the model with the lowest BIC is preferred [43]. Probability functions are closely related to the AIC [44].

Based on residuals, Steiger-Lind's RMSEA [45], RMSR [46], and SRMR [47] are all calculated. Excellent, good, and average fits are represented by RMSEA values of 0.01, 0.05, and 0.08, respectively [47].

Tests that compare the target model with the null model or relative/incremental indices exist. Examples include Bentler-Bonett's normative fit index (NFI) [48], non-normed fit index (NNFI) [48], Tucker-Lewis index (TLI) [49], comparative fit index (CFI) [50], relative fit index (RFI) [51], Parsimonious fit index (PNFI) [52], and incremental fit index (IFI). To demonstrate a sufficient fit, NFI/NNFI, TLI, CFI, RFI, PNFI, and IFI should be close to or above 0.90 [47].

Predictive fit indices or goodness-of-fit measurements from information theory are included, such as the Akaike information criterion (AIC) [53], the Joreskog goodness-of-fit index (GFI) [53], and the Joreskog's goodness-of-fit index adjusted (GFIA) [53] are the three different kinds of goodness-of-fit indices.

We also performed multi-group CFA using the entire sample to look at the questionnaire's sex invariance (i.e., males vs females) [54,55]. Metric and scalar levels of measurement invariance were used [54,55]. In behavioral research (including the present study), it has often been asked whether multiple-item scales can be represented as a one-factor model [56]. The conventional fit indices including RMSEA, SRMR, CFI, and others described above are excellent fit indices that are often used in factor analysis frameworks [56]. In recent years, conventional fit indices have been criticized [[56], [57], [58]]. Because these fit indices are continuous measures, the interpretation of their values is open to interpretation [[56], [57], [58]]. Thus, the dynamic fit index (DFI) cutoffs approach was introduced as a simulation study based on a fitted model designed and executed by an algorithm; mathematical details about the algorithm are available elsewhere [56]. To further strengthen our validation approach, in addition to conventional fit indices, we used the DFI approach.

### 2.2 Participants

A total of 811 persons made up the primary sample for the study. Adult status, possession of a mobile phone, and study participation were required as inclusion criteria. The exclusion criteria included refusal to cooperate with the investigation or a history of known mental illness. The sample was split into two equal parts, where 400 participants were used in the questionnaire development. The remaining 411 participants were used for questionnaire validation. A total of 811 participants participated, the mean age was 24.65 ± 05.66 (95%CI 24.26; 25.04); and 504 (62%) were female.

### 2.3 Ethical considerations

Prior to completing the questionnaire, all participants signed an electronic informed consent form. The study's objective, its confidential nature, and its anonymous data processing were explained. The Helsinki Guidelines were followed in terms of ethical principles. The Research Ethics Committee of Bahrain's Ministry of Health reviewed and authorized the study (MOH/114/June 22, 2022) in conformity with the fundamental ethical standards for research involving human beings outlined in the Declaration of Helsinki. The Guidelines for Responsible Data Management in Scientific Research were followed when managing data.

### 2.4 Data analysis

R version 4.2.2, and R Studio version 2022.02.0 were used to process the data. Sociodemographic factors were examined first (mean, standard deviation, frequencies, and percentages). A 95% confidence interval (95%CI) was created when appropriate. An independent sample *t*-test was conducted to compare the scores of males and females on the questionnaire. The level and direction of the link between several GHOST questionnaire items were evaluated using the Pearson product-moment correlation coefficient. Utilizing CVI, content validity was examined.

To evaluate factorability prior to conducting an EFA, Kaiser–Meyer–Olkin (KMO) and Bartlett's test of sphericity were computed [30]. The EFA and reliability calculation were conducted using a correlation matrix [59]. A Scree plot with parallel analysis was used to determine how sufficient factors should be extracted [32]. EFA was computed through an MLE method using no rotation [31]. CFA was performed using MLE with no standardization [60]. The package “dynamic” was used to compute DFI cutoffs [61]. The DFI is a metric used to assess how much the factor structure has changed over time based on the sequence of answers [56,57,62]. Higher values of the DFI, which has a range from 0 to 1, indicate better stability in the factor structure across time or between groups. Perfect invariance, or a DFI value of 1, denotes that the factor structure is constant through time or across groups [56,57,62]. Depending on the unique research issue and the study's context, DFI cutoffs must be interpreted [56,57,62]. However, there are some general guidelines for interpreting DFI cutoffs: A DFI value of 0.95 or higher is generally considered to indicate strong invariance, meaning that the factor structure is highly stable across time [56,57,62]. A DFI value between 0.90 and 0.95 suggests moderate invariance, meaning that there are some minor differences in the factor structure across time, but the overall structure is still relatively stable [56,57,62]. A DFI value below 0.90 indicates weak invariance, meaning that there are some minor differences in the factor structure across time. A statistically significant value was considered to be p < 0.05.

## 3 Results

### 3.1 Descriptive results

For the entire GHOST questionnaire, the mean score of the final version containing eight items was 21.74 ± 4.87 (95%CI 21.40; 22.07). Males and females had similar scores of 21.60 ± 4.90 and 21.82 ± 4.85, respectively p = 0.60. Table 1 shows the mean, standard deviation, and inter-correlation of the GHOST items. Correlation coefficients generally showed medium correlation size.

**Table 1:** Mean, standard deviation, and inter-correlation of the GHOST items (N = 811).

Variable	M	SD	Ghost 1	Ghost 2	Ghost 3	Ghost 4	Ghost 5	Ghost 6	Ghost 7	Ghost 8	Ghost 9	Ghost 10
Ghost 1	2.49	1.01	–									
Ghost 2	2.55	0.95	0.36*	–								
Ghost 3	2.80	0.92	0.22*	0.31*	–							
Ghost 4	2.63	1.05	0.14*	0.24*	0.30*	–						
Ghost 5	2.40	1.17	0.20*	0.25*	0.10*	0.23*	–					
Ghost 6	2.79	1.07	0.13*	0.18*	0.31*	0.28*	0.37*	–				
Ghost 7	2.73	1.03	0.09*	0.34*	0.36*	0.40*	0.21*	0.25*	–			
Ghost 8	2.82	0.99	0.17*	0.25*	0.24*	0.18*	0.21*	0.23*	0.40*	–		
Ghost 9	2.85	1.11	0.24*	0.28*	0.24*	0.21*	0.24*	0.19*	0.33*	0.28*	–	
Ghost 10	2.62	1.14	0.13*	0.31*	0.33*	0.19*	0.27*	0.25*	0.36*	0.22*	0.30*	–

Note: M = mean, SD = standard deviation, *p < 0.001. Correlation coefficients are the Pearson product-moment correlation coefficient. Ghost 1 (Neglect), Ghost 2 (Avoidance), Ghost 3 (Tardiness), Ghost 4 (Ambiguity), Ghost 5 (Blocking), Ghost 6 (Barriers), Ghost 7 (Absence), Ghost 8 (Inconsistently), Ghost 9 (Vulnerability), Ghost 10 (Withdrawal).

### 3.2 Reliability analysis results

The 811 participants were used to assess the unidimensional reliability. For the preliminary (10 items) version of the questionnaire the Cronbach's α, Ordinal α, and McDonald's ω were: 0.77 (95% 0.74; 0.79), 0.84 (95% 0.81; 0.87), 0.77 (95% 0.75; 0.79). The average inter-item correlation was 0.25 (95% 0.21; 0.29). For the final (8 items) version of the questionnaire the Cronbach's α, Ordinal α, and McDonald's ω were: 0.74 (95% 0.70; 0.76), 0.80 (95% 0.76; 0.82), 0.74 (95% 0.70; 0.75). Table 2 shows the individual item reliability statistics if an item is deleted and the item-rest correlation for both versions of the questionnaire. According to Table 1, a statistically significant correlation of p < 0.001 was observed between all the questionnaire items, suggesting acceptable convergent validity.

**Table 2:** Frequentist individual item reliability statistics if an item is deleted and item-rest correlation (N = 811).

The preliminary version of the Ghosting Questionnaire including 10-items				
Item	Cronbach's α	Ordinal α	McDonald's ω	Item-rest correlation
Overall	0.77	0.84	0.77	–
Ghost 1	0.76	0.83	0.76	0.31
Ghost 2	0.74	0.82	0.74	0.49
Ghost 3	0.75	0.81	0.75	0.46
Ghost 4	0.75	0.82	0.75	0.41
Ghost 5	0.75	0.82	0.76	0.40
Ghost 6	0.75	0.82	0.75	0.42
Ghost 7	0.74	0.81	0.74	0.53
Ghost 8	0.75	0.82	0.75	0.42
Ghost 9	0.75	0.83	0.75	0.44
Ghost 10	0.75	0.82	0.75	0.45
The final version of the Ghosting Questionnaire including 8-items				
Item	Cronbach's α	Ordinal α	McDonald's ω	Item-rest correlation
Overall	0.74	0.80	0.75	–
Ghost 1	0.76	0.83	0.76	0.31
Ghost 3	0.75	0.81	0.75	0.46
Ghost 4	0.75	0.82	0.75	0.41
Ghost 6	0.75	0.82	0.75	0.42
Ghost 7	0.74	0.81	0.74	0.53
Ghost 8	0.75	0.82	0.75	0.42
Ghost 9	0.75	0.83	0.75	0.44
Ghost 10	0.75	0.82	0.75	0.45

Note: Ghost 1 (Neglect), Ghost 2 (Avoidance), Ghost 3 (Tardiness), Ghost 4 (Ambiguity), Ghost 5 (Blocking), Ghost 6 (Barriers), Ghost 7 (Absence), Ghost 8 (Inconsistently), Ghost 9 (Vulnerability), Ghost 10 (Withdrawal).

### 3.3 EFA results (The preliminary version of the ghosting questionnaire 10-items)

**Fig. 2:**
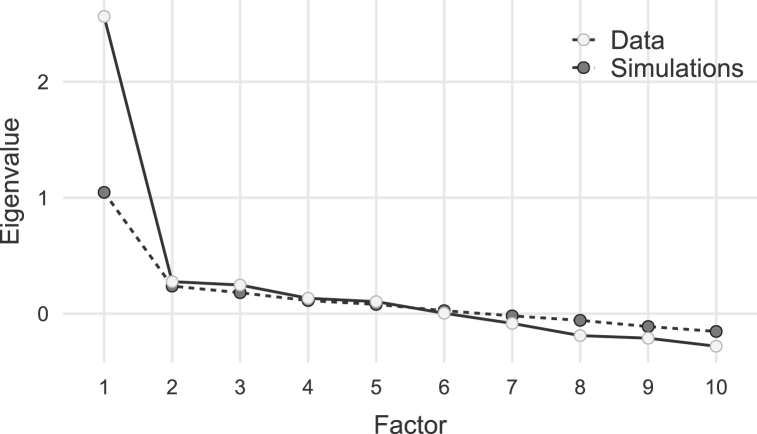
Scree plot of exploratory factor analysis.

The first 400 participants were included in an EFA to investigate the construct validity of GHOST and determine the factor structure of the questionnaire. Assumption checks showed that EFA is highly applicable to Bartlett's test of sphericity χ^2^ (df) = 715 (45), p < 0.001. KMO measure of sampling adequacy was 0.81 (ranging from 0.72 to 0.87). Results of EFA showed that all 10 items of the GHOST loaded under one single factor, with an eigenvalue of 2.56 and an average uniqueness of 0.75. Table 3 shows the factor loading after exploratory factor analysis. Fig. 2 provides a scree plot as a visual aid to determine the number of factors to retain in our EFA [63]. The scree plot demonstrated a difference between the eigenvalues of the kept factors and those of the non-retained factors, indicating that our questionnaire was best served by a one-factor solution explaining about 30% of the variance.

**Table 3:** Factor loading from exploratory factor analysis (N = 400).

Item	Loading	Uniqueness
Ghost 7	0.68	0.54
Ghost 3	0.56	0.69
Ghost 10	0.53	0.72
Ghost 2	0.52	0.72
Ghost 4	0.50	0.75
Ghost 9	0.49	0.76
Ghost 8	0.48	0.77
Ghost 5	0.46	0.79
Ghost 6	0.46	0.79
Ghost 1	0.34	0.89

Note: ‘Maximum likelihood estimation’ extraction method was used in combination with no rotation. Ghost 1 (Neglect), Ghost 2 (Avoidance), Ghost 3 (Tardiness), Ghost 4 (Ambiguity), Ghost 5 (Blocking), Ghost 6 (Barriers), Ghost 7 (Absence), Ghost 8 (Inconsistently), Ghost 9 (Vulnerability), Ghost 10 (Withdrawal).

According to the EFA results, two item pairs (Ghost 2 vs. Ghost 10 and Ghost 5 vs. Ghost 6) may not be adding unique variation to the measurement of the target construct because they had equal factor loadings and levels of uniqueness. These two pairs of items were consequently recognized as potential candidates to be eliminated from the instrument. Based on theoretical judgment and discussion among authors guided by the parsimony principle [64], which states that simpler models are favored over more complex ones when they explain the same amount of variance, these components were eliminated [64]. More specifically, the questionnaire items Ghost 2 (i.e., Despite sending messages, you have not heard back from them) and Ghost 5 (i.e., Have you been blocked or deleted from their social media apps or messaging apps) appeared to have similar loading and uniqueness with items Ghost 10 (i.e., They are not interested in meetings) and Ghost 6 (i.e., Have you experienced trouble maintaining a conversation with them (e.g., they use one-word replies, confusing emojis, short responses), respectively. Details are available in Table 3.

### 3.4 CFA results (The final version of the ghosting questionnaire 8-items)

The remaining 411 participants were included in a CFA to confirm the dimensionality and the validity of GHOST. Results of CFA showed that both baseline and factor models were statistically significant with χ^2^ (df) = 507 (28), p < 0.001, and χ^2^ (df) = 57 (20), p < 0.001, respectively. Table 4 shows the factor loading as a result of a CFA after excluding items Ghost 2 and Ghost 5.

**Table 4:** Factor loading from confirmatory factor analysis (N = 411).

Indicator	Estimate	Std. Error	z-value	p-value	95% LL	95% UL
Ghost 1	0.27	0.06	4.77	<0.001	0.16	0.39
Ghost 3	0.50	0.05	10.30	<0.001	0.41	0.60
Ghost 4	0.55	0.06	9.83	<0.001	0.44	0.66
Ghost 6	0.49	0.06	8.38	<0.001	0.37	0.60
Ghost 7	0.70	0.05	13.19	<0.001	0.60	0.81
Ghost 8	0.52	0.05	9.66	<0.001	0.42	0.63
Ghost 9	0.55	0.06	9.40	<0.001	0.44	0.67
Ghost 10	0.57	0.06	9.62	<0.001	0.45	0.69

Note: ‘Maximum likelihood estimation’ estimator method was used. Ghost 1 (Neglect), Ghost 2 (Avoidance), Ghost 3 (Tardiness), Ghost 4 (Ambiguity), Ghost 5 (Blocking), Ghost 6 (Barriers), Ghost 7 (Absence), Ghost 8 (Inconsistently), Ghost 9 (Vulnerability), Ghost 10 (Withdrawal).

Fit indices indicated highly fit results with CFI = 0.92; TLI = 0.90; NNFI = 0.90; NFI = 0.89; PNFI = 0.64; RFI = 0.85; IFI = 0.93; RNI = 0.93. The RMSEA = 0.07; RMSEA 90% CI lower bound = 0.05; RMSEA 90% CI upper bound = 0.08; SRMR = 0.04; Hoelter's critical N α = 0.05 = 230; GFI = 0.99; MFI = 0.96; ECVI = 0.25. Details are provided in Table 5. Metric and scalar invariance models suggest no difference between males and females. Specifically, estimates were 1.21 and 1.20 with p < 0.001 for metric and scalar factor variances, respectively.

**Table 5:** Fit indices from confirmatory factor analysis (N = 411).

Index	Value
Chi-square test = 56.489	p < 0.001
Comparative Fit Index (CFI)	0.92
Tucker-Lewis Index (TLI)	0.89
Bentler-Bonett Non-normed Fit Index (NNFI)	0.89
Bentler-Bonett Normed Fit Index (NFI)	0.89
Parsimony Normed Fit Index (PNFI)	0.64
Bollen's Relative Fit Index (RFI)	0.84
Bollen's Incremental Fit Index (IFI)	0.93
Relative Noncentrality Index (RNI)	0.92
Root mean square error of approximation (RMSEA)	0.07
RMSEA 90% CI lower bound	0.05
RMSEA 90% CI upper bound	0.09
RMSEA p-value	0.08
Standardized root mean square residual (SRMR)	0.04
Hoelter's critical N (α = .05)	229.53
Hoelter's critical N (α = .01)	274.32
Goodness of fit index (GFI)	1.00
McDonald fit index (MFI)	0.96
Expected cross validation index (ECVI)	0.25

Note: ‘Maximum likelihood estimation’ estimator method was used. Ghost 1 (Neglect), Ghost 2 (Avoidance), Ghost 3 (Tardiness), Ghost 4 (Ambiguity), Ghost 5 (Blocking), Ghost 6 (Barriers), Ghost 7 (Absence), Ghost 8 (Inconsistently), Ghost 9 (Vulnerability), Ghost 10 (Withdrawal).

**Fig. 3:**
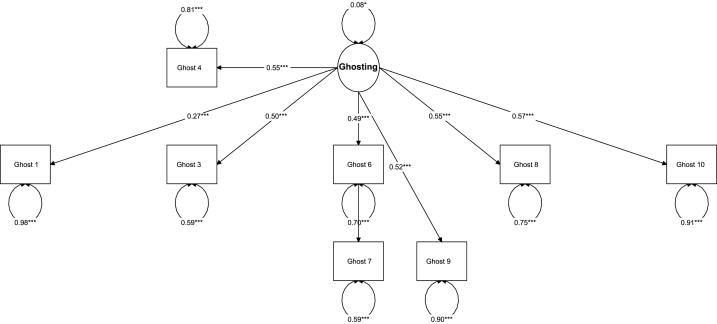
Path diagram of the Ghost Questionnaire.

**Fig. 4:**
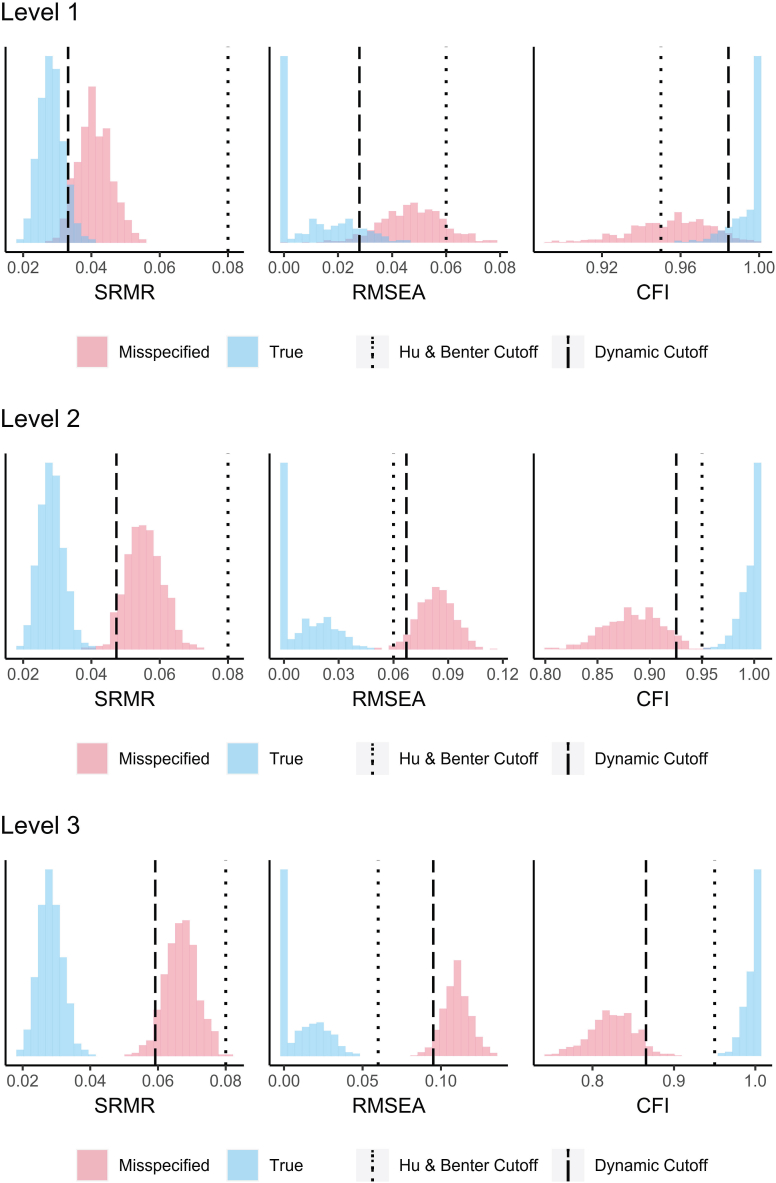
Visual results of dynamic fit index (DFI) cutoffs. Level 1: The fitted model plus a 0.30 residual correlation between one-third of the items. Level 2: The fitted model plus a 0.30 residual correlation between two-thirds of the items. Level 3: The fitted model plus a 0.30 residual correlation between all of the items.

Fig. 3 provides the path diagram of the Ghost Questionnaire. Results of DFI cutoffs also showed high dynamic fit = Level 1: 90/10 SRMR = 0.03; RMSEA = 0.03; CFI = 0.98. Level 2: 95/5 SRMR = 0.05; RMSEA = 0.06; CFI = 0.97 Level 3: 95/5 SRMR = 0.06; RMSEA = 0.09; CFI = 0.95. Visual results are shown in Fig. 4.

## 4 Discussion

Ghosting is a phenomenon that has become increasingly common in today's society. It is a form of abandonment in which a person suddenly stops all contact with another person without explanation or warning. This can be done through social media, phone calls, emails, and text messages. The act of ghosting can be extremely upsetting for the victim and leave them feeling bewildered, disappointed, and angry. Ghosting is fundamentally an emotional control technique. By abruptly ending communication, the individual who ghosts another person manipulates their emotions. Ghosting may be used to punish someone or to avoid dealing with a challenging circumstance. The victims of ghosting might not always get an explanation or resolution. There has not been any empirical research on ghosting, even though it has received a lot of media attention [65].

Due to the growing interest in the topic, it is important to develop a new questionnaire to advance research and improve our understanding of ghosting as a psychological and behavioral construct.

The ghosting questionnaire developed in this initially contained 10 items: Ghost 1 (Neglect), Ghost 2 (Avoidance), Ghost 3 (Tardiness), Ghost 4 (Ambiguity), Ghost 5 (Blocking), Ghost 6 (Barriers), Ghost 7 (Absence), Ghost 8 (Inconsistently), Ghost 9 (Vulnerability), Ghost 10 (Withdrawal).

Through the assessment of the dimensionality of the construct through EFA and parallel analysis, it was observed that the suggested model presented only one factor. In EFA the solution suggested removing items that lacked the appropriate psychometric characteristics, which yielded a final questionnaire version comprising eight items. The two items removed were Ghost 2 (Avoidance) and Ghost 5 (Blocking).

Subsequently, CFA of the questionnaire with eight items was carried out and showed good fitness with all items having adequate factor loadings. CFA exhibited invariance by sex, implying that the factor structure and item loadings are equivalent for both males and females. Therefore, the questionnaire items appear to be measuring the same construct in males and females.

The initial 10 items and final eight item versions of the questionnaire have similar reliability indices. The relationship between the number of items and the reliability of a questionnaire is complex and depends on several factors. In general, having more items on a scale can improve its reliability, but there are also some potential disadvantages to consider. The deleted two items appeared to be redundant, and they did not impact the questionnaire's internal consistency.

### 4.1 Implications to practice

A questionnaire to assess ghosting was developed for several reasons: First, ghosting is a new phenomenon that is still not completely understood. Researchers can gain a better understanding of the prevalence, causes, and effects of ghosting on people and relationships by creating a tool to measure it [13,17]. Second, a questionnaire to quantify ghosting can assist healthcare providers in evaluating patients who are suffering distress linked to ghosting as ghosting can have major detrimental effects on health and well-being [3,19,66]. Third, ghosting is a complex activity that can take place in a range of settings and relationships. Researchers and therapists can better understand the various causes of ghosting behavior and develop strategies for prevention and intervention by using a measure to quantify ghosting [67]. Fourth, creating a questionnaire to estimate ghosting can help us gain a better understanding of how social media and technology are altering how we interact with one another and build connections [20,68,69].

### 4.2 Implications for future research

A ghosting questionnaire has several advantages. First, it can help researchers better understand ghosting behavior and its effects in different types of relationships. By measuring the frequency and characteristics of ghosting, researchers are able to find patterns and trends to guide interventions and assistance. Another benefit of a ghosting questionnaire is that it can provide insights into the psychological and communicative processes that lead to ghosting. Studying variables such as attachment type, emotion control, and communication patterns can help researchers better understand the causes and experiences of ghosting. As a third benefit, ghosting questionnaires are an excellent tool for improving understanding of one's own or other's dating-related behaviors. The results of standardized questionnaires can reveal people's habits and preferences and those of others. As a result, relationships can be managed better, communication can be improved, and ghosting experiences can be handled better. A ghosting questionnaire might prove useful for academics, professionals, and people interested in understanding and coping with ghosting in various types of relationships. Through providing standardized and adaptable measures to deal with ghosting experiences, our questionnaire can contribute to a better understanding of different types of relationships and improve well-being for those who are affected by ghosting.

The individual who is ghosted may experience a variety of undesirable consequences. Feelings of abandonment, bewilderment, and melancholy may result [4,6,9]. The individual may become afraid of intimacy or closeness in the future, which can undermine trust in relationships [4,6,9]. This may necessitate psychological assistance as it might lead to resentment and unhappiness [4,6,9]. The best method for a victim of ghosting to cope with the situation is to make an effort to comprehend why it occurred and to realize how frequently it occurs.

This can be difficult, as the person who involved in ghosting someone may not be willing to explain their motives. However, it is important, when counseling a ghosting victim, to emphasize that ghosting reflects on the perpetrator and not on the ghostee's worth or desirability. Our questionnaire has successfully captured the essence of ghosting and provides a working definition of the term: refusing to communicate or refusing to maintain communication with a previous contact via technical means without explanation either sudden or gradual. Any relationship is subject to ghosting, although romantic relationships appear to be ghosted more frequently than others [1,5].

Generally, the ghostee is not aware that they are being purposely ghosted. This is important as our questionnaire can be used as a self-assessment measure to determine whether or not one's communications are no longer wanted [5,6]. The questionnaire can also be used to inquire about various aspects of relationships that end in ghosting [70]. Recent research showed that ghosting can be common in the workplace [15]. The study used 554 participants to evaluate workplace ghosting by analyzing the influence of feedback (or lack thereof) on applicants' psychological needs fulfillment. Although the present study found no indication that being ghosted by a possible employer makes candidates feel worse than being outright rejected, such an experience can still be painful, and additional research is needed to understand the long-term impacts of being ghosted by a potential employer [15].

### 4.3 Strengths and limitations

This study has several strengths. The development of the ghosting questionnaire involved a thorough review of the literature on the topic of ghosting, which helped to ensure that the questionnaire covers relevant items. The use of a qualitative method to identify and generate questionnaire items is a rigorous approach that can improve the content validity of the questionnaire. The research involved a large sample size, which enhances the generalizability of the findings. The psychometric testing of the ghosting questionnaire indicated that it is a valid and reliable instrument for measuring ghosting experiences, as demonstrated by good content and construct validity, acceptable internal consistency, and robust fitting.

It should be noted that although the validity and reliability of the designed instrument were confirmed, this study had its limitations. These limitations need to be considered in future research to refine and improve the questionnaire. One limitation is that the EFA had a low score for the total percentage of explained variance. As a result, this might indicate that additional investigation and interpretation are required to completely comprehend the underlying structure of the data.

This validation study is based on the Classic Theory Test (CTT), and we have not performed Item Response Theory (IRT). Thus, we suggest that research perform IRT to provide some additional important information, i.e., item difficulty, discriminative ability, etc. [71]. Additionally, future research needs to establish the Minimum Clinical Important Difference (MCID) [72] of this instrument which will facilitate its intervention application.

Invariance analysis was not performed among all subgroups. Testing the degree to which the factor structure is constant across other groups, such as various sexes or cultures, is known as an invariance analysis in factor analysis [41]. Aiming to ensure that any reported changes in factor scores are not the result of variations in the factor structure itself, invariance analysis examines whether the same underlying factor structure is present across various groups. Future research needs to consider multiple variables for invariance analyses e.g., age, sex, education level, marital status, income, etc.

Moreover, the ghostee experience is measured by the instrument, but the ghoster experience is not. The act of ghosting may have negative effects as well – guilt, feelings of disloyalty, and feelings of cowardice. Furthermore, test-retests were not conducted to assess the instrument's temporal stability. Because this is a new tool, it was not possible to compare its psychometric properties to other instruments. Convenience samples are vulnerable to selection bias, as researchers may select participants who are more likely to provide the desired outcome. Thus, we suggest that future researchers use an improved sampling framework e.g., simple random samples. The study was conducted in English; therefore, unless it is translated, its application is limited to English speakers. Performing CFAs in future studies would be beneficial to ensure construct validity in different samples. It is hoped that this questionnaire will lead to preventive and curative interventions for the distress occasioned by this new form of social distancing and abandonment.

A thorough and flexible questionnaire was provided by our study to assess the construct of ghosting in the context of relationships. Although our results offer insightful information about the prevalence and effects of ghosting, future studies should consider computing normative scores to further validate and improve the measurement characteristics of our questionnaire. By using normative scores, we may better understand the distribution and variability of answers and pinpoint particular populations or subgroups that would be more likely to encounter or engage in ghosting behavior. We anticipate that our research will advance knowledge of the dynamics of relationships and aid in the creation of efficient interventions and support systems for ghosting victims.

## 5 Conclusions

The GHOST questionnaire was developed to examine the experience of ghosting and proved to be valid and reliable. A psychometric analysis of the questionnaire revealed that it has sufficient content validity, construct validity, and internal consistency to be used in further investigations of this modern phenomenon.

## Funding

This research received no external funding.

## Ethical approval/institutional review board statement

The Research Ethics Committee of Bahrain's Ministry of Health reviewed and authorized the study (MOH/114/June 22, 2022) in conformity with the fundamental ethical standards for research involving human beings outlined in the Declaration of Helsinki.

## Informed consent statement

Informed consent was obtained from all subjects involved in the study.

## Author contribution statement

Haitham Jahrami; Zahra Saif: Conceived and designed the experiments; Performed the experiments; Analyzed and interpreted the data; Contributed reagents, materials, analysis tools or data; Wrote the paper.

Wen Chen; Mai Helmy; Hadeel Ghazzawi; Khaled Trabelsi; Gabriel Natan Pires; Nicola L. Bragazzi; Seithikurippu R. Pandi-Perumal; Mary V. Seeman.

## Data availability statement

Data will be made available on request.

## Declaration of competing interest

The authors declare that they have no known competing financial interests or personal relationships that could have appeared to influence the work reported in this paper.
