# Supplementary material for: Development and validation of a questionnaire (GHOST) to assess sudden, unexplained communication exclusion or “ghosting"
Source: Heliyon. 2023 Jun 8;9(6):e17066. doi: 10.1016/j.heliyon.2023.e17066 (PMC10361225; doi:10.1016/j.heliyon.2023.e17066)
Supplement: Multimedia component 1 [file mmc1.docx]

**The Ghosting Questionnaire (The preliminary version)**

Instructions: Ghosting, is best defined when someone abruptly ends all communication with another person without warning.

The majority of us associate it with the digital withdrawal of a friend who doesn't text back, or worse—a lover—but it actually occurs in all kinds of social contexts (e.g., work connections) and is influenced by how we perceive the world.

Please answer the following questions based on your personal experience of being "ghosted" over the past six months.

Report the most common behaviors you have experienced. The pronouns they/them/theirs refer to the "ghosters".

| Questions | Always =5 | Often = 4 | Sometimes =3 | Rarely = 2 | Never =1 |
| --- | --- | --- | --- | --- | --- |
| 1. Did you get stood up or had your plans canceled by them without being told beforehand**.** |  |  |  |  |  |
| 1. Despite sending messages, you have not heard back from them**.** |  |  |  |  |  |
| 1. Their reply/response messages are delayed**.** |  |  |  |  |  |
| 1. Their reply/response messages are confusing and vague**.** |  |  |  |  |  |
| 1. Have you been blocked or deleted from their social media apps or messaging apps**.** |  |  |  |  |  |
| 1. Have you experienced trouble maintaining a conversation with them (*e.g.,* they use one word replies, confusing emojis, short responses)**.** |  |  |  |  |  |
| 1. The phrase "I'm busy" is always used in their communications**.** |  |  |  |  |  |
| 1. Their interest in you is inconsistent sometimes very engaged, sometimes completely uninterested**.** |  |  |  |  |  |
| 1. They don't share personal information about themselves with you**.** |  |  |  |  |  |
| 1. They are not interested in meeting**.** |  |  |  |  |  |

**Note:** Permissions to use the Ghosting Questionnaire (GHOST) are granted on a single use, and per study.

For permissions contact Dr. Haitham Jahrami [haitham.jahrami@outlook.com](mailto:haitham.jahrami@outlook.com)

**The Ghosting Questionnaire (The final version)**

Instructions: Ghosting, is best defined when someone abruptly ends all communication with another person without warning.

The majority of us associate it with the digital withdrawal of a friend who doesn't text back, or worse—a lover—but it actually occurs in all kinds of social contexts (e.g., work connections) and is influenced by how we perceive the world.

Please answer the following questions based on your personal experience of being "ghosted" over the past six months.

Report the most common behaviors you have experienced. The pronouns they/them/theirs refer to the "ghosters".

| Questions | Always =5 | Often = 4 | Sometimes =3 | Rarely = 2 | Never =1 |
| --- | --- | --- | --- | --- | --- |
| 1. Did you get stood up or had your plans canceled by them without being told beforehand**.** |  |  |  |  |  |
| 1. Their reply/response messages are delayed**.** |  |  |  |  |  |
| 1. Their reply/response messages are confusing and vague**.** |  |  |  |  |  |
| 1. Have you been blocked or deleted from their social media apps or messaging apps**.** |  |  |  |  |  |
| 1. The phrase "I'm busy" is always used in their communications**.** |  |  |  |  |  |
| 1. Their interest in you is inconsistent sometimes very engaged, sometimes completely uninterested**.** |  |  |  |  |  |
| 1. They don't share personal information about themselves with you**.** |  |  |  |  |  |
| 1. They are not interested in meeting**.** |  |  |  |  |  |

**Note:** Permissions to use the Ghosting Questionnaire (GHOST) are granted on a single use, and per study.

For permissions contact Dr. Haitham Jahrami [haitham.jahrami@outlook.com](mailto:haitham.jahrami@outlook.com)
